# Fundus Autofluorescence in Multiple Evanescent White Dot Syndrome

**DOI:** 10.1155/2011/807565

**Published:** 2011-09-21

**Authors:** Fernando Marcondes Penha, Eduardo Vitor Navajas, Fábio Bom Aggio, Eduardo B. Rodrigues, Michel Eid Farah

**Affiliations:** Department of Ophthalmology, Vision Institute (IPEPO), Federal University of São Paulo, 04021-001 São Paulo, SP, Brazil

## Abstract

A patient complained of photopsia and vision loss in the left eye for two days, with visual acuity of 20/32. Right eye was normal. Funduscopy revealed foveal granularity and gray-white lesions in the posterior pole, mainly temporal to the fovea. The lesions (dots and spots), along with a few other areas surrounding them, showed hyperautofluorescence on autofluorescence imaging. Fluorescein angiogram (FA) depicted some early hyperfluorescent dots with late staining. Indocyanine green angiogram (ICGA) showed hypofluorescent lesions in a greater number compared with funduscopy, autofluorescence, and FA. Thirty days later, BCVA was 20/20 in both eyes and the complimentary exams were almost normal, despite an ICGA that showed few small hypofluorescent lesions. This case supports the hypothesis that the choroidal involvement occurs primarily in MEWDS, with secondary involvement of the RPE and the neurosensory retina.

## 1. Introduction

Multiple evanescent white dot syndrome (MEWDS) is an idiopathic intraocular inflammatory disorder characterized by transient small white dot fundus lesions first reported in 1984 [1]. Some important aspects of the disorder remain controversial, for instance the precise nature and initiating process of the associated fundus lesions as well as their angiographic characteristics. Autofluorescent imaging of the ocular fundus relies on the stimulated emission of light from molecules in the retinal pigment epithelium (RPE), sign of previous and possible future, and oxidative injury [2]. In this report we present the angiographic and autofluorescence findings of a patient with MEWDS.

## 2. Case Report

**Figure 1:**
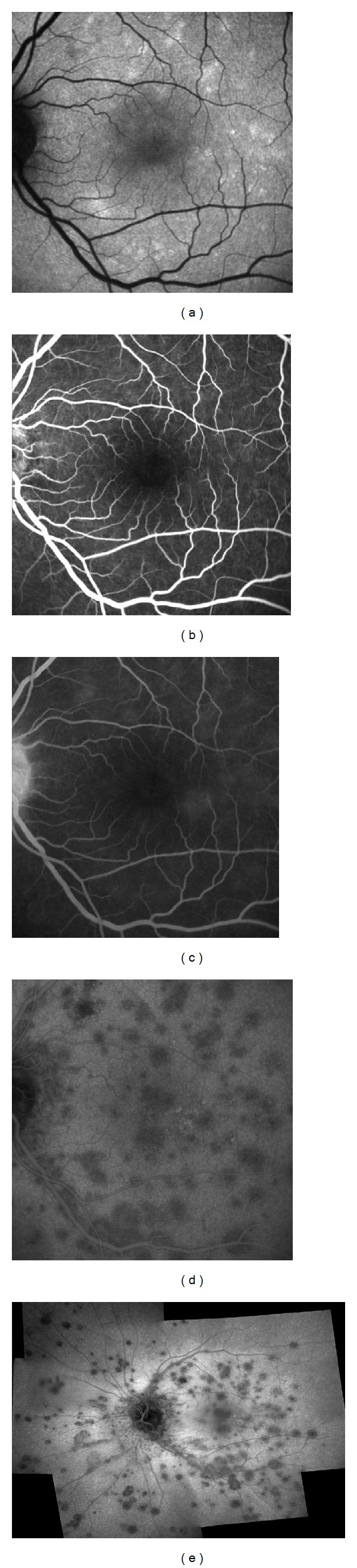
Initial presentation. (a) Fundus autofluorescence showing hyperfluorescent dots in macular area. (b) Early frame of fluorescein angiogram (FA) shows hyperfluorescent dots. (c) Late frame FA shows enlargement of hyperfluorescent lesion. (d) Late frame indocyanine green angiogram reveals hypofluorescent lesions. (e) Panoramic reconstruction of ICGA showing a great number of hypofluorescent lesions.

**Figure 2:**
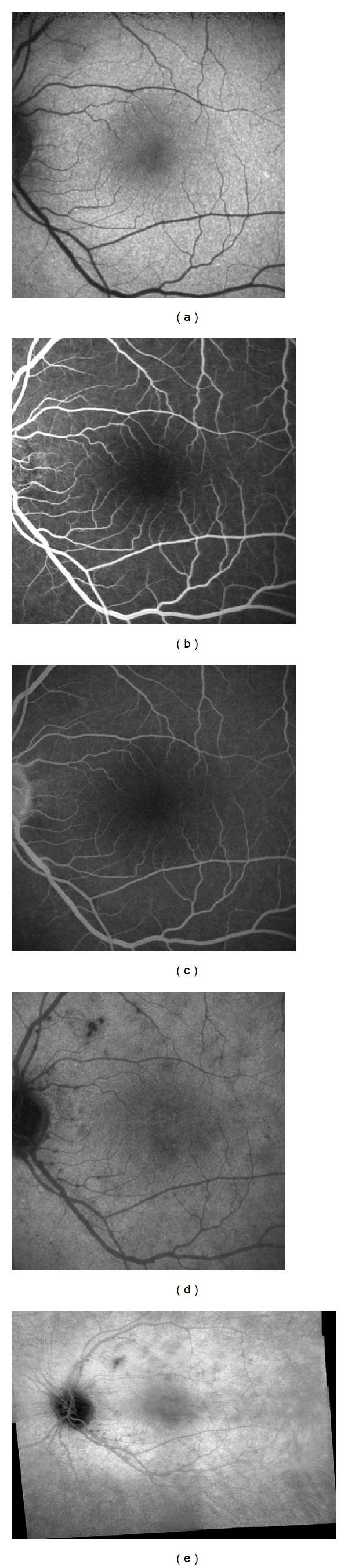
Thirty-day follow-up visit. (a) Normal fundus autofluorescence exam. (b) Early frame of normal fluorescein angiogram (FA). (c) Late frame FA with no hyperfluorescent lesion. (d) Late frame indocyanine green angiogram reveals hypofluorescent lesions mainly in peripapillary area. (e) Panoramic reconstruction of ICGA showing small hypofluorescent lesions.

A 32-year-old white woman complained of photopsia and vision loss in the left eye (OS) for two days. Best-corrected visual acuity (BCVA) was 20/20 in the right eye and 20/32 in the OS. Funduscopy revealed foveal granularity and gray-white lesions in the posterior pole, mainly temporal to the fovea. These dots and spots lesions along with a few other areas surrounding them showed hyperautofluorescence on autofluorescence imaging (Figure 1(a)). Fluorescein angiogram (FA) depicted some early hyperfluorescent dots (Figure 1(b)) with late staining (Figure 1(c)). Indocyanine green angiogram (ICGA) showed hypofluorescent lesions (Figures 1(d) and 1(e)) in a greater number compared with funduscopy, autofluorescence, and FA. Thirty days later patient recalled sporadic episodes of photopsia, BCVA was 20/20 in both eyes. Autofluorescence exam was almost normal, as well as FA. ICGA showed few small hypofluorescent lesions (Figure 2).

## 3. Discussion

Clinical manifestations of MEWDS have been described in the retina, the choroid, and the optic nerve, including transient white dot fundus lesions (100–200 *μ*m), macular granularity, and mild inflammation of the optic nerve [3]. The inflammatory disease is suspected to be the result of a viral infection, possibly with an immune-mediated mechanism in a genetically susceptible person, but its precise pathogenesis remains unknown [4]. The ophthalmologic literature suggests that the evanescent lesions in MEWDS are located in the RPE and outer retina [5]. This assumption is based on their clinical and angiographic appearance and on electrophysiological evidence, which has demonstrated an electro-oculographic reduction in the light-dark ratio, as well as electroretinographic alteration of the a-wave and early receptor potential. Nevertheless, some studies emphasize that choroidal involvement occurs first, as hypofluorescent lesions seen on indocyanine green angiogram may appear even in normal areas on fluorescein angiogram and funduscopy [5]. Our case supports the hypothesis that the choroidal involvement occurs primarily in MEWDS, with secondary involvement of the RPE and the neurosensory retina. In the areas in which the inflammatory infiltrates from the choroid reach the RPE, its function becomes impaired and the accumulation of lipofuscin granules takes place, leading to the hyperautofluorescent lesions herein demonstrated.

## References

[1] Jampol LM, Sieving PA, Pugh D (1984). Multiple evanescent white dot syndrome. I. Clinical findings. *Archives of Ophthalmology*.

[2] Spaide RF (2003). Fundus autofluorescence and age-related macular degeneration. *Ophthalmology*.

[3] Bryan G, Freund KB, Yannuzzi LA, Spaide RF, Huang SJ, Costa DL (2002). Multiple evanescent white dot syndrome in patients with multifocal choroiditis. *Retina*.

[4] Jampol LM, Becker KG (2003). White spot syndromes of the retina: a hypothesis based on the common genetic hypothesis of autoimmune/inflammatory disease. *American Journal of Ophthalmology*.

[5] Gross NE, Yannuzzi LA, Freund KB, Spaide RF, Amato GP, Sigal R (2006). Multiple evanescent white dot syndrome. *Archives of Ophthalmology*.

